# First insights into the miRNA landscape of *Tursiops truncatus* milk reveal shared dominant microRNA families with terrestrial mammals

**DOI:** 10.1371/journal.pone.0356241

**Published:** 2026-09-09

**Authors:** Filippo Cendron, Carlo Boselli, Flavio Maggi, Annalisa Duri, Valentina D’Onofrio, Massimo De Marchi, Mauro Penasa, Umberto Rosani

**Affiliations:** 1 Department of Agronomy, Food, Natural Resources, Animals and Environment, University of Padova, Legnaro, Italy; 2 Istituto Zooprofilattico Sperimentale del Lazio e della Toscana “M. Aleandri” - National Reference Centre for Ovine and Caprine Milk and Dairy Products Quality (C.Re.L.D.O.C.), Rome, Italy; 3 Azienda Sanitaria Locale, Roma 4, Distretto 4, Rignano Flaminio, Rome, Italy; 4 General practitioner freelance AniCura - Clinica Veterinaria Borghesiana, Rome, Italy; 5 Department of Biology, University of Padova, Padova, Italy; MARE – Marine and Environmental Sciences Centre, PORTUGAL

## Abstract

MicroRNAs (miRNAs) are small non-coding RNAs that play crucial regulatory roles in gene expression in metazoans. While the miRNA repertoire and relative abundances have been extensively studied in terrestrial mammals, no information was available for the milk of marine mammals. Here, we present the first characterization of the miRNA genomic landscape and abundance in milk in the bottlenose dolphin (*Tursiops truncatus*). Using a sequence-based comparative approach, we identified 186 conserved miRNA families comprising 354 high-confidence precursors in the dolphin genome. Comparative analysis across 52 cetacean genomes revealed a small number of lineage-specific loss events, such as *mir-187* in Delphinidae, and the absence of nine miRNA families in all cetaceans. Small RNA sequencing from pooled milk samples confirmed the detectable abundance of 119 miRNAs, with a landscape dominated by *mir-148*, *let-7*, *mir-8*, and *mir-21*, collectively accounting for over 70% of total miRNA reads. These dominant families include miRNAs frequently reported in the milk of terrestrial mammals, suggesting qualitative similarity in the major milk miRNA repertoire between dolphin and terrestrial mammals. These findings should be interpreted as a first sequencing-supported exploratory characterization of dolphin milk miRNAs.

## Introduction

MicroRNAs (miRNAs) are conserved small, non-coding RNAs that post-transcriptionally act on gene expression by binding target messenger RNAs (mRNAs) [1]. Since their discovery over 30 years ago [2], miRNAs have emerged as key regulators in biological processes, including development, cell differentiation, immune response, metabolism, and disease [3]. Based on conserved sequence features dictated by the highly controlled processes of miRNA biogenesis and function, miRNAs can be distinguished from other short RNAs or RNA degradation products present in cells [4–6]. Likely because of their relevant regulative roles, many miRNAs have followed precise evolutionary paths, with genomic gain and loss events often associated with phenotypic traits or species-specific adaptations in metazoans, as evidence by an increasing number of miRNA families reported from basal metazoans to mammals [7,8]. miRNAs have now been identified in almost all metazoan species. A growing body of research is focused on conserved expression patterns of miRNAs in analogous organs, underpinning possible regulative functions conserved across evolution. This is exemplified by *mir-51* in *Caenorhabditis. elegans*, which is part of the *mir-100* family present in all eumetazoans, and functions via adding regulatory complexity to cell signalling [9]. Beyond intracellular functions, miRNAs can also be released into extracellular fluids, where they are often stabilized by RNA-binding proteins, lipoproteins, or are encapsulated within extracellular vesicles such as exosomes [10,11]. The presence of miRNAs has been reported in mouse mammary gland cell lines [12] and in breast milk [13], free in the medium or packaged into extracellular vesicles [14]. This extracellular population of miRNAs has gathered increasing attention for the potential roles in intercellular communication and, particularly in the context of maternal–offspring communication, for the potential to promote molecular signaling through milk feeding. Human and bovine milk have been shown to contain a diverse repertoire of miRNAs, several of which are shared across species and are thought to play roles in neonatal immune system maturation, intestinal development, and metabolic programming [10,13,15,16]. So far, the majority of miRNA research in milk has focused on terrestrial mammals, and no information on the diversity or biological significance of miRNAs in the milk of marine mammals is currently available. Cetaceans, including dolphins, represent a unique clade of marine mammals that have undergone extensive evolutionary adaptations to the aquatic environment, including changes in physiology, sensory biology, metabolism, and reproduction [17,18]. In particular, lactation in cetaceans exhibits several specialized features, such as the composition of milk, the structure of the mammary gland, and the duration and energetics of maternal investment, all of which may be reflected in the molecular cargo of milk, including its miRNA content [19]. The bottlenose dolphin (*Tursiops truncatus*) is a protected species and one of the best-studied cetaceans, due to their accessibility and presence in managed care settings [20]. Despite the relative ease of obtaining dolphin biological samples, so far only a single study has characterized the miRNA profile in dolphin’s tissues (raw data not available) [21].

To expand current knowledge of dolphin miRNAs, we carried out the first characterization of miRNAs in the milk of *T. truncatus*. First, a genome-wide identification of miRNA families was undertaken on the *T. truncatus* reference genome, and conservation with other cetaceans was evaluated. Subsequently, milk samples obtained at multiple lactation stages were analyzed to characterize the miRNA abundance profile. These findings advance our understanding of the dolphin miRNA repertoire and provide a framework for future studies of shared and lineage-specific miRNA profiles across mammalian milk samples.

## Results

### Comparative annotation of the *T. truncatus* miRNA landscape across cetaceans

**Fig 1:**
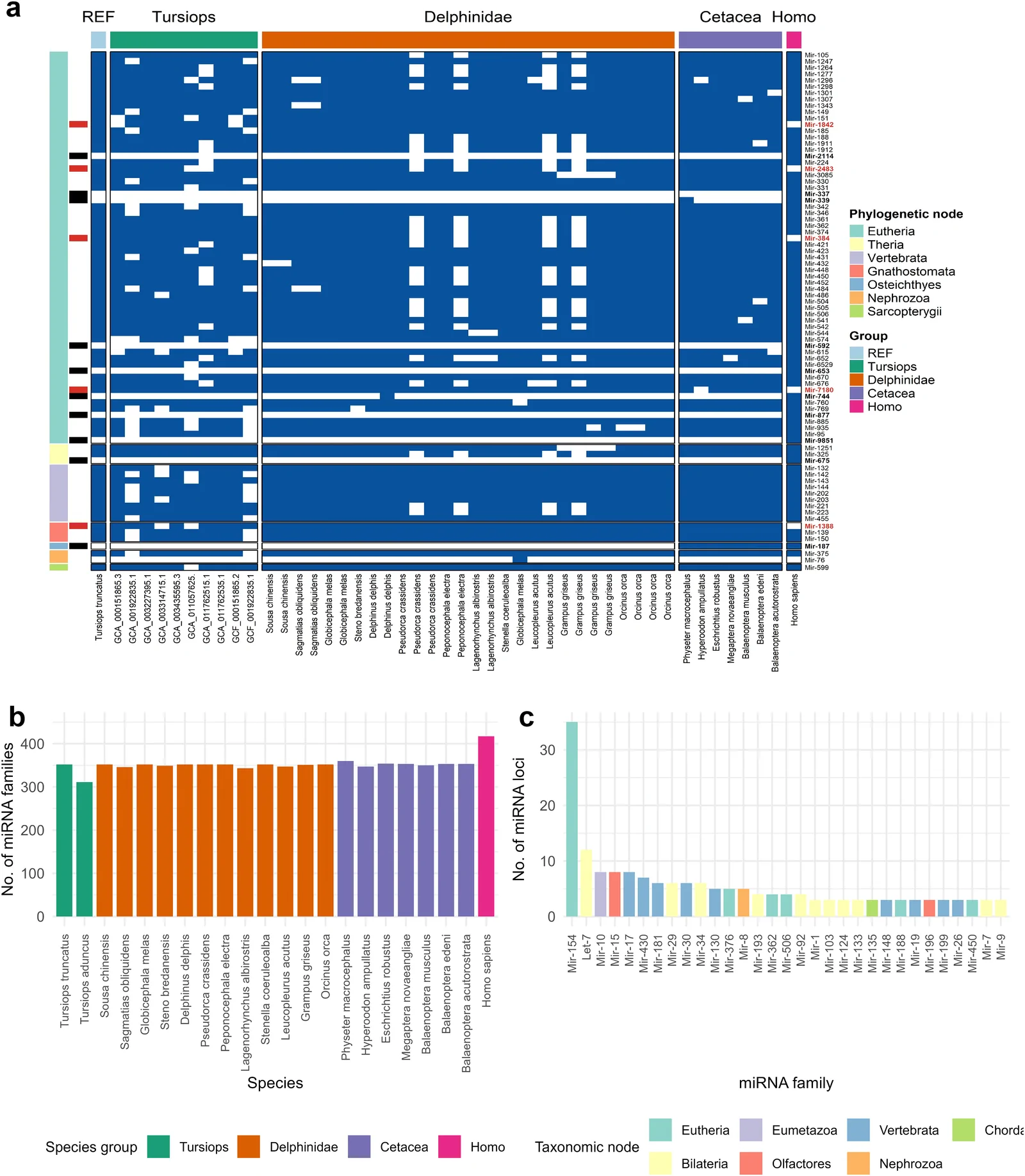
miRNA landscape in the *Tursiops truncatus* genome and comparative presence/absence across cetaceans. a. Presence/absence heatmap of 220 taxonomically informed miRNA families across cetacean and *H. sapiens* genomes. miRNA families are grouped according to their phylogenetic node of origin, indicated by the coloured side annotation. Analysed genomes are grouped on the X-axis as the *T. truncatus* reference genome (named ‘REF’), other *Tursiops* genomes, Delphinidae, other Cetacea, and *H. sapiens*. White boxes indicate absence and dark-blue boxes indicate presence of the corresponding miRNA family. miRNA family names highlighted by bolded-black coloured text indicate putative loss events, whereas names highlighted in red coloured text indicate miRNA families detected in cetaceans but absent from *H. sapiens*. b. Total number of detected miRNA families in each analysed genome. Bars are coloured according to the same taxonomic group scheme used in panel a. c. Number of miRNA loci per miRNA family in the *T. truncatus* reference genome. Only miRNA families with more than two detected loci are shown. Bars are coloured according to the phylogenetic node of origin of each miRNA family.

Using a sequence-based approach we tested the *T. truncatus* reference genome for the presence of 220 taxonomically informed miRNA families, expected to be either present (189) or lost (31) in the group of placental mammals (Eutheria). Approximately 90% of the families thought to be present were identified and all the lost miRNA families were absent (S1 Table). Notably, the absence of some Eutherian miRNA families suggested either genome incompleteness, genuine loss events in *T. truncatus* or a combination of both. To further investigate this aspect we analyzed the patterns of presence/absence of the 220 miRNA families in all the genome assemblies of cetaceans (S1 Table). This analysis revealed the loss of *mir-187*, which was consistently absent in all Delphinidae species but present in other cetaceans (Fig 1a). Nine additional miRNA families (*mir-2114*, *mir-337*, *mir-339*, *mir-592*, *mir-653*, *mir-675*, *mir-744*, *mir-877* and *mir-9851*) were also determined to be lost from all cetaceans. On the other hand, *mir-1388*, *mir-1842*, *mir-2483*, *mir-384* and *mir-7180* were present in most cetacean assemblies (>80% in each of the tested groups, namely “Tursiops”, “Delphinidae” or “other cetaceans”), whereas they were absent in humans according to both our analysis and mirGeneDB [22]. Overall, the number of predicted miRNA families appeared to be consistent among cetacean genomes, ranging from 311 to 360, compared with 413 miRNA families predicted to be present in the human genome (Fig 1b).

**Fig 2:**
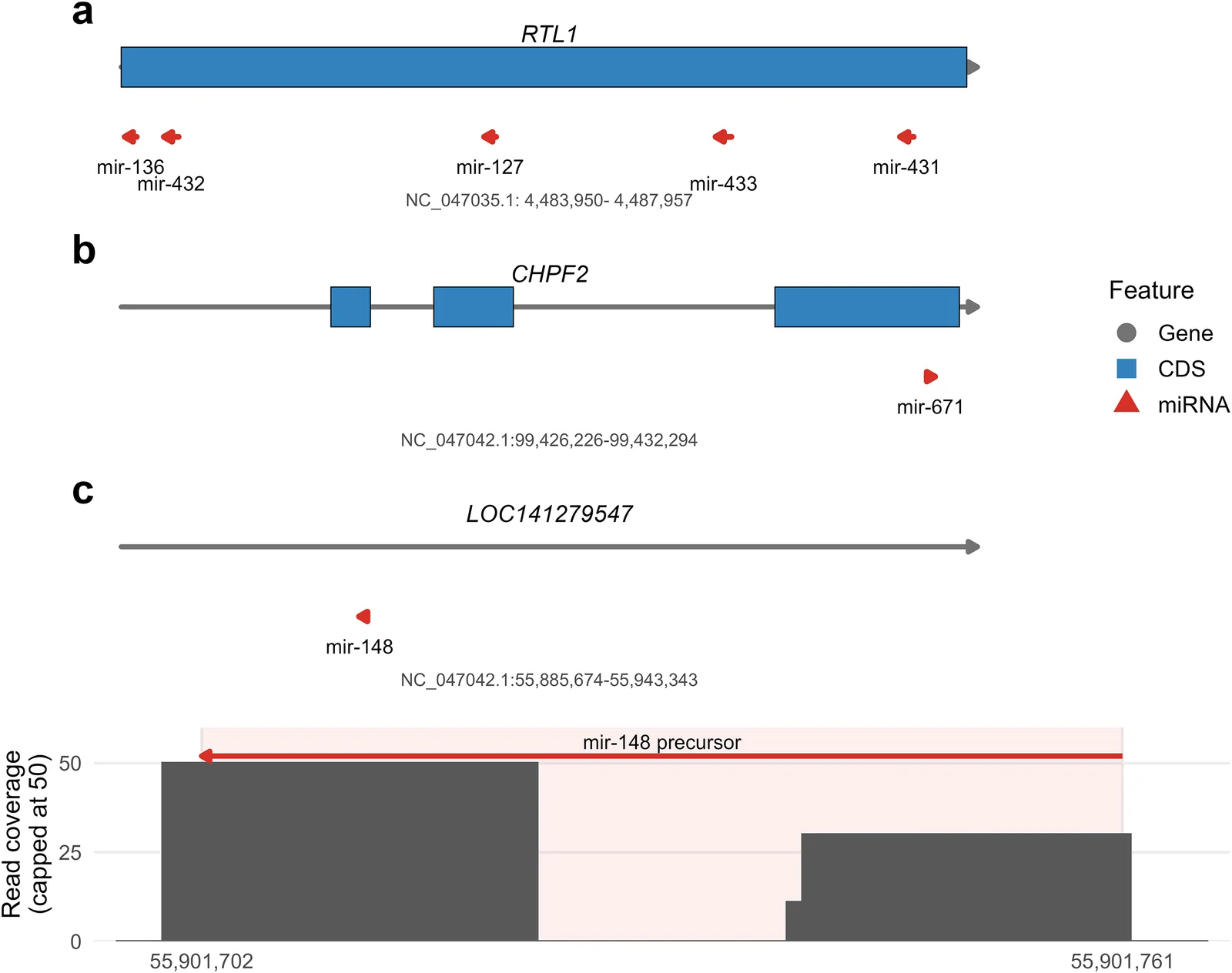
Representative genomic organization of selected miRNA loci in the *Tursiops truncatus* genome. a. *RTL1*-associated miRNA cluster, including mir-127, mir-136, mir-431, mir-432, and *mir-433*. b. *CHPF2*-overlapping mir-671 locus. The miRNA precursor overlaps a coding sequence of chondroitin sulfate glucuronyltransferase (*CHPF2*). c. Genomic context of the most abundant milk miRNA locus, *mir-148.PRE_NC_047042.1_55901702_55901761*, located within the uncharacterized gene *LOC141279547*. d. Magnified view of the *mir-148* precursor region ±5 bp showing small RNA-seq read coverage across the annotated precursor. Coverage values are capped at 50 reads for visualization to make both precursor-arm signals visible. Arrow direction indicates strand orientation. Genomic coordinate ranges below panels a–c correspond to the reference-genome coordinates used to generate the scaled locus diagrams.

A second round of analysis was performed on the *T. truncatus* reference genome by use of 732 metazoan miRNA families as queries, thus including also non-mammalian families. Overall, we identified 186 families with 354 miRNA precursors, which were predicted with high confidence on the *T. truncatus* genome, with over 17,693 total predictions, mostly referring to low confidence hits (S1 File). With this second analysis we could add seven miRNA families to the ones identified using only Eutheria-specific miRNA families, namely *mir-1007, mir-967* and *mir-303* (found in *D. melanogaster*), *mir-2* and *mir-36* (protostomes), *mir-2387* and *mir-370* (boreoeutheria). Only the latter two miRNA families were expected to be present in dolphins, since they belong to the same taxonomic node. Notably, all these seven miRNA families are present with a single locus each. In contrast, multiple miRNA loci were detected for *mir-154* (N = 35), *let-7* (N = 12), *mir-10*, *mir-1*5 and *mir-17* (N = 8) (Fig 1c). A total of 210 hits referring to 170 miRNAs were determined to have intragenic position (59% of the total identified), with fourteen other hits corresponding to nine additional miRNAs determined to be positioned in the coding sequence (CDS) of a protein coding gene.. The latter group included *mir-1306*, whose precursor sequences overlap with those which encode the microprocessor gene, *DGCR8*, a genic structure known to be conserved among different animal species, and which is involved in a negative-feedback regulation of *DGCR8* via suboptimal microprocessor substrates [23,24]. Similarly, the overlap of *mir-985* locus with the *CACNG8* locus has been already reported in mice and humans [25,26]*.* Representative genomic examples are shown in Fig 2. The cluster including *mir-127, mir-136, mir-431, mir-432*, and *mir-433* was located antisense to the *RTL1* gene locus, consistent with a conserved eutherian genomic organization (Fig 2a) [27]. A further example was represented by *mir-671*, which overlapped the coding sequence of *CHPF2*, a gene which encodes a protein involved in chondroitin sulphate biosynthesis (Fig 2b). Finally, the precursor encoding the sequence of the most abundant miRNA detected in the milk sequencing dataset, *mir-148* (precursor named PRE_NC_047042.1_55901702_55901761), mapped within an uncharacterized gene region (Fig 2c), and the magnified coverage plot showed read support over both annotated precursor arms (Fig 2d). Together, these examples illustrate both conserved miRNA–protein coding gene architectures and read-supported miRNA loci in the *T. truncatus* genome.

### The miRNA abundance profile in milk revealed shared dominant miRNA families with terrestrial mammals

A pooled milk sample collected from two female dolphins at different lactation stages (N = 76 samples, S1 Fig) was used for small RNA library preparation and sequencing (sRNA-seq). A complete read-processing and mapping summary is provided in S2 Table. Briefly, high-throughput sRNA sequencing generated 45.6 million raw reads. After adapter removal and quality trimming, 8.9 million reads were retained, of which 1.54 million were within the 18–24 nt range and were used for miRNA analysis. Among trimmed reads, 7.9 million were classified as tRNA-derived reads, explaining the prominent 33-nt peak observed in the size-distribution analysis (S2a Fig). Of the 18–24 nt reads, 89.84% mapped to the *T. truncatus* genome and 24.41% mapped to predicted miRNA precursors, showing a typical 22-nt peak (S2b Fig). Reads assigned to high-confidence miRNA predictions accounted for 98.8% of all reads mapping to predicted miRNA sequences. Genomic unmapped reads showed a much more random and continuous size distribution (S2c Fig).

A total of 119 miRNAs were labelled as ‘present’, defined as miRNAs with read counts exceeding the arbitrary cut-off of 100 mapped reads (representing a value referring to <0.03% of the miRNA reads), whereas none of the miRNAs classified as non-eutherian reached this minimal abundance cutoff (S3 Table). The top 20 accumulating miRNAs included 75.58% of the reads and are reported in Table 1 by abundance. For each of these abundant miRNAs, Table 1 also reports whether the same miRNA family has previously been described in milk from terrestrial mammals and summarizes putative regulatory associations inferred from published studies. Most of the dominant dolphin milk miRNA families, including *mir-148, let-7, mir-21, mir-26, mir-29, mir-101, mir-30*, and *mir-15*, have been reported in milk from at least one other mammalian species, whereas the functional annotations mainly relate to lipid metabolism, immune regulation, mammary physiology, oxidative-stress response, and developmental processes (see Table 1 for references).

**Table 1:** Abundance levels of the most accumulating miRNAs in dolphin milk. Relative abundance percentages were calculated as the proportion of reads assigned to each miRNA precursor relative to the total number of reads mapped to high-confidence miRNA precursors. The miRNA ID, miRNA family, relative abundance calculated as percentage of mapped reads over total reads mapped on high-confidence precursors, species in which the same miRNA family has been reported in milk, and reported regulatory associations in other mammalian systems are reported. The miRNA ID includes genomic coordinates.

miRNA ID (our work)	miRNA family	Relative abundance (%)	Conservation in milk	Reported regulatory associations in other mammalian systems
Mir-148.PRE_NC_047042.1_55901702_55901761	*mir-148*	19.09	Human, cow, goat, donkey, and buffalo	Involved in fat and lipid metabolism, and immune response [28]
Let-7.PRE_NC_047044.1_3723722_3723798	*let-7*	9.54	Human, cow, goat, sheep, porcine, donkey, and buffalo	Milk production, pregnancy biomarker and immune response [29,30]
Mir-8.PRE_NC_047044.1_95840375_95840439	*mir-8*	8.29	Human	Hyperthermia‐induced lactate secretion [30]
Mir-8.PRE_NC_047034.1_183475063_183475123	*mir-8*	4.72	Human	Hyperthermia‐induced lactate secretion
Mir-21.PRE_NC_047053.1_29623608_29623667	*mir-21*	4.35	Human, mice, cow, goat, and donkey	Development of the immune and inflammatory response [31]
Mir-26.PRE_NC_047040.1_19211982_19212038	*mir-26*	3.01	Human, cow, and goat	Fat synthesis regulation and immune response [31–33]
Mir-29.PRE_NC_047042.1_83822078_83822137	*mir-29*	2.76	Human, cow, goat, donkey, and buffalo	Resistance to heat stress and milk production traits [31,34–36]
Let-7.PRE_NC_047044.1_3724678_3724747	*let-7*	2.73	Human, cow, goat, sheep, porcine, donkey, and buffalo	Milk production, pregnancy biomarker and immune response
Let-7.PRE_NC_047039.1_27549403_27549475	*let-7*	2.69	Human, cow, goat, sheep, porcine, donkey, and buffalo	Milk production, pregnancy biomarker and immune response
Let-7.PRE_NC_047041.1_10986486_10986552	*let-7*	2.48	Human, cow, goat, sheep, porcine, donkey, and buffalo	Milk production, pregnancy biomarker and immune response
Mir-101.PRE_NC_047034.1_128236819_128236877	*mir-101*	1.99	Human, cow, and goat	Cell proliferation and oxidative stress-induced apoptosis [37,38]
Mir-101.PRE_NC_047039.1_60526183_60526241	*mir-101*	1.95	Human, cow, and goat	Cell proliferation and oxidative stress-induced apoptosis [37,38]
Mir-30.PRE_NC_047034.1_151011645_151011708	*mir-30*	1.91	Cow, sheep	Immune response and fat metabolism [39]
Mir-29.PRE_NC_047034.1_36190620_36190677	*mir-29*	1.87	Human, cow, goat, donkey, and buffalo	Inflammation and oxidative stress and regulating milk production traits [40]
Mir-30.PRE_NC_047045.1_8619526_8619588	*mir-30*	1.86	Cow, sheep	Immune response and fat metabolism [39]
Mir-29.PRE_NC_047034.1_36067397_36067454	*mir-29*	1.85	Human, cow, goat, donkey, and buffalo	Inflammation and oxidative stress and regulating milk production traits
Let-7.PRE_NC_047055.1_83413024_83413095	*let-7*	1.45	Human, cow, goat, sheep, porcine, donkey, and buffalo	Milk production, pregnancy biomarker and immune response
Mir-15.PRE_NC_047051.1_17540826_17540890	*mir-15*	1.35	Human, cow	Lipid metabolism and pregnancy biomarker and immune response [41,42]

**Fig 3:**
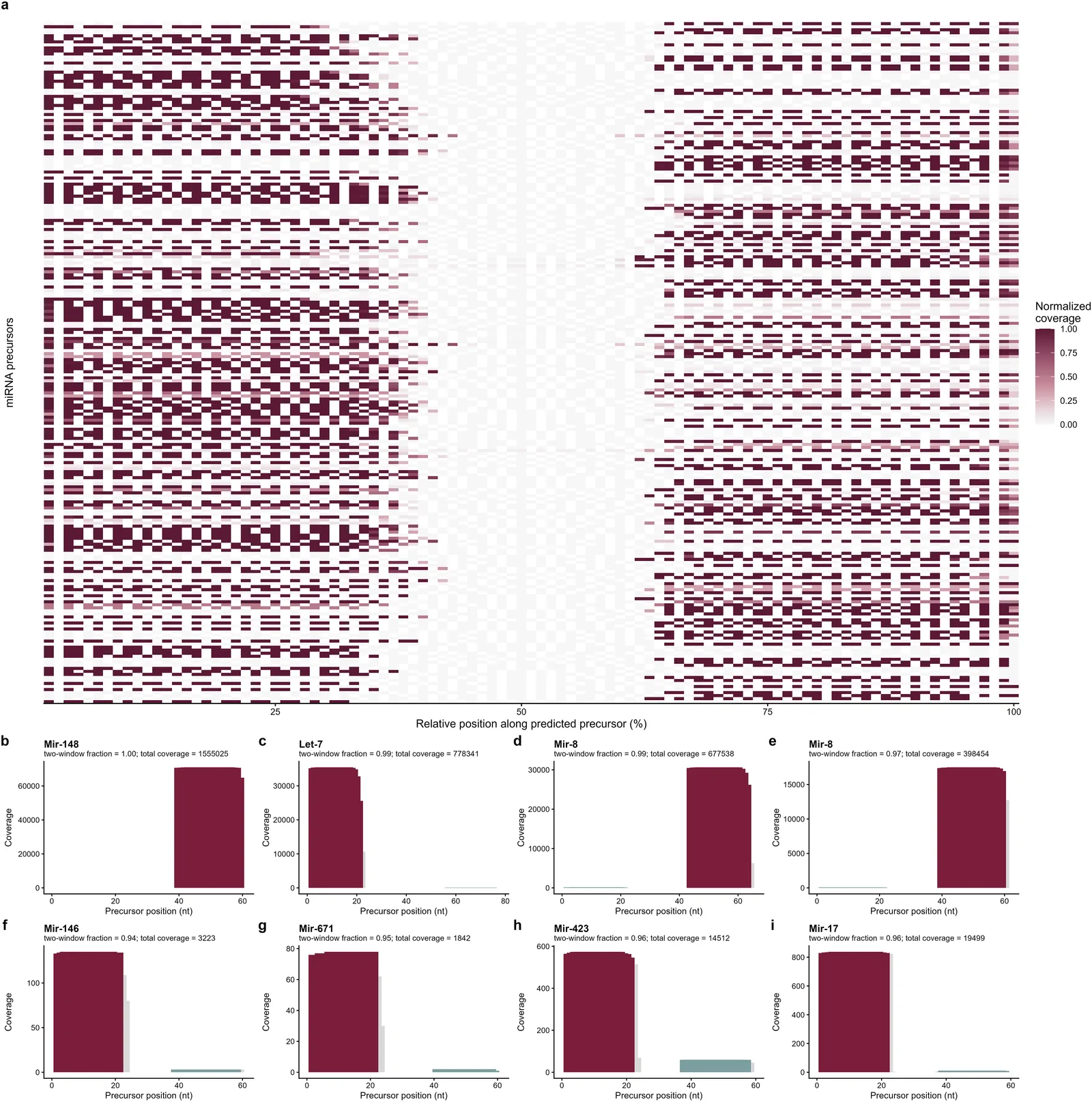
Positional distribution of small RNA-seq coverage across high-confidence miRNA precursors. a. Heatmap showing per-position coverage across the 354 high-confidence predicted miRNA precursors. Precursor lengths were scaled to 100 relative-position bins, and coverage was normalized to the maximum value within each precursor. b-i. Per-position coverage profiles for eight representative precursors. The primary and secondary non-overlapping 22-nt windows with the greatest cumulative coverage are shown in burgundy and muted teal, respectively, whereas coverage outside these intervals is shown in grey. The two-window fraction indicates the proportion of total base coverage contained within the two selected windows.

Notably, coverage analysis performed on the 354 high-confidence precursor sequences showed that sRNA-seq coverage was generally concentrated in one or two discrete regions of approximately mature-miRNA length, rather than being uniformly distributed across the full precursor sequence (Fig 3a and S4 Table). The global heatmap revealed a recurrent pattern of localized coverage near one or both ends of the predicted precursors, with comparatively limited coverage across the central region. Representative highly supported precursors displayed one strongly dominant region and, in some cases, a second lower-abundance region, with the two highest-coverage non-overlapping 22-nt windows accounting for most of the total precursor coverage (Fig 3b-i). These patterns are consistent with preferential accumulation of processed small RNAs from discrete precursor regions.

## Discussion

In this study, we combined a genome-wide characterization of miRNAs in the *T. truncatus* genome with sRNA-seq data from a pooled milk small RNA-seq dataset covering different lactation stages. Owing to the near-complete absence of genomic location and abundance data for miRNAs in dolphin species, we first performed a comparative characterization of miRNA families using mirGeneDB [22] and MirMachine [43]. For the most part, the miRNA landscape in *T. truncatus* followed the expected presence/absence family patterns, except for specific cases, which may represent genuine loss events in Delphinidae or cetacean species. Theoretically, miRNA presence/absence data can support the taxonomic classification of species [22,44]. This may be relevant for oceanic dolphins because Delphinidae has sometimes been considered a taxonomically complex family, with several phylogenetic relationships still under active debated [45]. However, in the present dataset, miRNA presence/absence patterns did not provide additional resolution for fine-scale relationships among the analyzed Delphinidae species, because the observed loss events were shared across all tested representatives. Losses of miRNA families are generally rare and often associated with peculiar phenotypic traits [46,47]. We can speculate that the loss of *mir-187* in Delphinidae might reflect differences in regulatory mechanisms during early development, since in humans it localizes in the placenta in early pregnancy and has been associated with recurrent miscarriage [48]. However, inhibition of *mir-187* resulted in cancer progression, suggesting that it can also be involved in control of tumour cell growth [49,50] and, as demonstrated for many other miRNAs, its pleiotropic role makes any speculation difficult. We observed nine missing miRNA families when comparing cetaceans with terrestrial mammals (using human as a proxy for the latter species), namely *mir-2114, mir-337, mir-339, mir-592, mir-653, mir-675, mir-744, mir-877* and *mir-9851*. Several of these miRNA families are involved in tumor progression or in inhibiting cell invasions. One intriguing observation, which would deserve dedicated studies, is the fact that marine mammals appear to have a lower cancer incidence than expected for their body size and lifespan. So far, this has been attributed to the positive selection and expansion of genes with cancer-protective functions [51], although the possible involvement of miRNA in this process might be considered in future studies.

Interestingly, moving from taxonomic-restricted miRNA families to all the miRNA families of metazoans did not substantially change the number of detected miRNAs, with the few additional hits not supported by abundance data. On the one hand, this highlights the importance of coupling miRNA predictions with sRNA-seq data. On the other hand, this finding supports the use of taxonomically informed miRNA families as phylogenetic markers or, as recently proposed, as quality markers for genome completeness [43]. Overall, the localization of some miRNAs within genes or CDS regions was consistent with previously described conserved miRNA/gene architectures across mammals [52].

Based on a single sample, obtained by pooling 76 milk samples covering two stages of dolphin lactation, we could provide sRNA-Seq based evidence of the accumulation of 119 miRNAs in milk out of the 354 bioinformatically identified in the dolphin genome. For all these precursors, the read distribution was consistent with localized processing from one or both precursor arms, with little or no coverage across the central region. The absence of accumulation of detectable levels for low-quality miRNA predictions further supports the reliability of the prediction workflow and suggests that only high-confidence predictions should be considered for further analyses. The most highly accumulated hits detected in our milk sample referred to miRNAs also identified in milk samples of terrestrial mammals. *Mir-148* was among the top accumulated miRNAs and has likewise been reported in human, cow, goat, donkey and buffalo milk [28]. Similarly, members of the *let-7* family were also determined to be highly abundant in our dataset and have been identified in milk from human, cow, goat, donkey, buffalo, pig and sheep [16,53,54]. In other mammalian systems, many of these miRNAs have been already studied in relation to offsprings. For example, *mir-148* is involved in fat and lipid metabolism and plays a role in immune response modulation, processes that are essential for normal development and maturation from early life to adulthood [55]. Likewise, *mir-101*, highly abundant in dolphin milk and reported in milk of terrestrial mammals, is implicated in the regulation of genes associated with cellular proliferation and plays a critical role in oxidative-stress responses [55,56]. Other miRNAs, such as *mir-29* and *mir-8*, have been associated with the regulation of milk-production-related genes in humans; however, *mir-8* has never been identified in milk of terrestrial species other than *H. sapiens*, and its function appears to be specifically linked to hyperthermia-induced lactate secretion [57]. Overall, these observations are consistent with shared abundance of several major milk miRNA families between terrestrial mammals and this marine species. However, because the analysis was based on one pooled dolphin milk library and a literature-based comparison rather than a formal cross-species analysis, the present data should not be interpreted as definitive evidence of conserved expression levels or conserved regulatory function.

## Conclusions

We provide the first genome-wide annotation of miRNA families in the *T. truncatus* genome and the first sRNA-seq-based characterization of miRNAs in bottlenose dolphin milk. Sequencing of a pooled milk RNA sample identified 119 miRNAs which accumulated to detectable levels and showed that several of the most abundant dolphin milk miRNA families, including *mir-148, let-7, mir-8*, and *mir-21*, are also frequently reported in milk samples of terrestrial mammals. These findings support qualitative similarity in dominant milk miRNA families between dolphin and terrestrial mammals, but they should be regarded as preliminary because they are based on a single pooled milk library, literature-based cross-species comparison, and no independent RT-qPCR validation due to the lack of residual RNA material. Future studies should include biological replication, stage-specific profiling, targeted validation of abundant miRNAs, and formal cross-species analyses to test whether these similarities reflect conserved regulatory mechanisms or lineage-specific features of cetacean lactation.

## Materials and methods

### Data retrieval

The *T. truncatus* reference genome was retrieved from the NCBI Genome database (ID: GCF_011762595.2) together with the available genomic annotations in gff format. Other genomes available for cetaceans were selected based on phylogeny [58] and were similarly retrieved (S5 Table).

### miRNA identification and annotation in the dolphin genomes

To annotate miRNAs in the *T. truncatus* genome we applied two rounds of MirMachine [43] analysis. The first was used to identify the presence or absence of taxonomically informed miRNA families, selecting the node “Eutheria”, setting the model for deuterostomia with the following command: *MirMachine.py --node Eutheria -a --species Tursiops_truncatus --genome GCF_011762595.2.fa*. This analysis was applied to all the selected genomes. The second round was applied only to the *T. truncatus* reference genome and was used to comprehensively annotate miRNA candidates (all metazoan miRNA families, N = 732) irrespective of the expected taxonomic information and resulted in a set of low- and high-confidence candidates. The resulting annotations in gff format were added to the genomic annotations as “microRNA” (S1 File).

### Milk sampling, RNA extraction and sncRNA sequencing

A total of 76 milk samples were collected from two dolphins housed at the marine park Zoomarine Italia Spa in Torvaianica, Pomezia (Rome, Italy). Both animals were multiparous females, aged between 10 and 12 years, maintained under identical environmental conditions and provided with the same standardized diet, consisting of capelin (*Mallotus villosus*), sprat (*Sprattus sprattus*), herring (*Clupea harengus*), mackerel (*Scomber scombrus*), horsemackerel (*Trachurus trachurus*), squid (*Loligo spp.*) and blue whiting (*Micromesistius poutassou*). Milk samples were collected at two distinct stages of lactation: early lactation from 1 to 14 months post-partum, and late lactation from 14 to 38 months post-partum (S1 Fig). Sampling was conducted through voluntary cooperation of the animals, with collections performed twice daily, once per week. During the procedure, the dolphins were trained to position themselves laterally, exposing the mammary slit above the water surface to allow access to milk collection. The calf was always present during sampling. Milk was obtained using sterile syringes specially adapted for this purpose. Collection was facilitated by creating negative pressure through gentle retraction of the syringe plunger. Immediately after collection, milk samples were refrigerated at 4 °C and subsequently transferred to a –80 °C freezer at the Istituto Zooprofilattico Sperimentale del Lazio e della Toscana “Mariano Aleandri” (Rome, Italy). Prior to RNA extraction, samples were thawed on ice and combined to prepare a pooled sample (50 mL), which was used for the sequencing and identification of miRNAs. Total RNA was extracted from 2 mL of milk matrix derived from a total pooled volume of 50 mL, using the exoRNeasy Matrix kit (Qiagen, Venlo, The Netherlands). A total of 100 ng of purified RNA was used to prepare miRNA libraries for high-throughput sequencing. Following adapter ligation, Unique Molecular Identifiers (UMI) were incorporated during the reverse transcription step. Complementary DNA (cDNA) was then amplified by PCR (16 cycles), and the resulting products were purified. Library quality was assessed via capillary electrophoresis using the Agilent TapeStation D1000 system (Agilent Technologies, Santa Clara, CA, USA) and an appropriate library quantity was sequenced using a 75 bp single-end read configuration (Illumina Inc., San Diego, CA, USA). The sequencing dataset has been deposited in the NCBI SRA archive under accession ID PRJNA1404193.

### Short non-coding RNA sequencing data analysis

Adapter trimming was performed with cutadapt [59] using the adapter sequence AACTGTAGGCACCATCAAT, retaining only reads with detected adapter sequence and applying a minimum PHRED quality threshold of 25. For global sncRNA size-distribution analyses, trimmed reads in the 18–35 nt range were retained. For miRNA annotation and abundance quantification, analyses were restricted to reads of 18–24 nt. sRNA reads were mapped to the *T. truncatus* reference genome using CLC Genomics Workbench v26.0 with similarity and length fractions set to 0.9. Reads overlapping annotated genes, CDS features, tRNA-derived regions, and newly annotated microRNA loci were counted. Size profiles for the mapped reads were created using R. Figures were generated in R using *readxl*, *dplyr*, and *tidyr* for data import and handling; *ComplexHeatmap*, *circlize*, *ggplot2*, and *ggpubr* for visualization; *RColorBrewer* for palette definition; and *grid* for graphical annotation.

### Ethical statement

Sample collection was performed exclusively during routine veterinary health monitoring and milk quality assessment procedures conducted by the facility’s veterinarians, Dr. Flavio Maggi and Dr. Annalisa Duri. These procedures were part of the regular management and welfare monitoring program aimed at evaluating milk quality and nutritional adequacy for nursing calves. No animals were handled, restrained, disturbed, or subjected to any additional procedures specifically for the purposes of this study. The research was conducted exclusively on biological material obtained during routine veterinary activities, and therefore no experimental intervention involving animals was performed. Animal care and management were carried out in accordance with the applicable national and institutional regulations governing animal welfare.

## Supporting information

S1 Table Genomic landscape of miRNAs in cetacean species. The table reports the results of the search for taxonomically informed miRNA families in the *T. truncatus* genome, in other 52 cetacean and in *H. sapiens* genomes for comparison. For the 220 miRNA families the node of origin is reported together with the number of hits in all genomes. Genomes have been divided into four groups, namely Tursiops, dolphins, cetaceans and humans.
(XLSX)

S2 Table Statistics for sncRNA data analysis. The table reported the number and percentages of read fractions associated with the different analytical steps.
(XLSX)

S3 Table Abundance analysis of dolphin miRNAs. The number of read counts is reported for the 17,681 miRNA predictions on the *T. truncatus* reference genomes.
(XLSX)

S4 Table Coverage metrics computed for the high-confidence miRNA precursors. For each precursor, the table reports the ID and length, total base coverage summed across all precursor positions, maximum per-position coverage, and the coordinates and cumulative coverage scores of the primary and secondary non-overlapping 22-nt windows with the highest coverage. f_primary indicates the proportion of total precursor base coverage contained within the primary window, whereas f_two_windows indicates the combined proportion contained within the primary and secondary windows. Precursors are ranked by decreasing total base coverage, and the corresponding miRNA family name is provided.
(XLSX)

S5 Table Metadata of the analyzed genomes. The assembly ID, name, sex of the sequenced individual, name of the species, assembly quality level, release date and number of scaffolds are indicated.
(XLSX)

S1 Fig Sampling of dolphin milk. (A) Device used for milk collection. (B) At the beginning of the milk-extraction procedure, the dolphin voluntarily positions herself laterally. (C) Initial aspiration phase with vacuum application to teat (the suction chamber of device begins to fill). (D) Progressive accumulation of milk in the device’s suction chamber is due to the effect of the applied vacuum. Photographs courtesy of Flavio Maggi and Annalisa Duri. Copyright retained by the authors.
(JPG)

S2 Fig Size distribution of small non-coding RNA reads. Density plots show the size distribution, in the 18–35 nt range, of a. tRNA-derived reads, b. reads mapped to predicted miRNA precursors, and c. genome-unmapped reads.
(TIFF)

S1 File miRNA annotation file including all miRNA predictions on the *T. truncatus* genome (gff format compatible with the reference genome, ID: GCF_011762595.2). (TXT)

## References

[1] Bartel DP. MicroRNAs: genomics, biogenesis, mechanism, and function. Cell. 2004;116(2):281–97. doi: 10.1016/s0092-8674(04)00045-5 14744438

[2] Lee RC, Feinbaum RL, Ambros V. The C. elegans heterochronic gene lin-4 encodes small RNAs with antisense complementarity to lin-14. Cell. 1993;75(5):843–54. doi: 10.1016/0092-8674(93)90529-y 8252621

[3] Krol J, Loedige I, Filipowicz W. The widespread regulation of microRNA biogenesis, function and decay. Nat Rev Genet. 2010;11(9):597–610. doi: 10.1038/nrg2843 20661255

[4] Russell SJ, Zhao C, Biondic S, Menezes K, Hagemann-Jensen M, Librach CL, et al. An atlas of small non-coding RNAs in human preimplantation development. Nat Commun. 2024;15(1):8634. doi: 10.1038/s41467-024-52943-w 39367016 PMC11452719

[5] Lopes I de ON, Schliep A, de Carvalho LF. The discriminant power of RNA features for pre-miRNA recognition. BMC Bioinformatics. 2014;15:124. doi: 10.1186/1471-2105-15-124 24884650 PMC4046174

[6] Ledda B, Ottaggio L, Izzotti A, Sukkar SG, Miele M. Small RNAs in eucaryotes: new clues for amplifying microRNA benefits. Cell Biosci. 2020;10:1. doi: 10.1186/s13578-019-0370-3 31911829 PMC6942390

[7] Dexheimer PJ, Cochella L. MicroRNAs: from mechanism to organism. Front Cell Dev Biol. 2020;8:409. doi: 10.3389/fcell.2020.00409 32582699 PMC7283388

[8] Hertel J, Lindemeyer M, Missal K, Fried C, Tanzer A, Flamm C, et al. The expansion of the metazoan microRNA repertoire. BMC Genomics. 2006;7:25. doi: 10.1186/1471-2164-7-25 16480513 PMC1388199

[9] Santillan EM, Cormack ED, Wang J, Rodriguez-Steube M, Cochella L. An ancient and essential miRNA family controls cellular interaction pathways in C. elegans. Sci Adv. 2025;11(36):eadz1934. doi: 10.1126/sciadv.adz1934 40901950 PMC12407057

[10] Valadi H, Ekström K, Bossios A, Sjöstrand M, Lee JJ, Lötvall JO. Exosome-mediated transfer of mRNAs and microRNAs is a novel mechanism of genetic exchange between cells. Nat Cell Biol. 2007;9(6):654–9. doi: 10.1038/ncb1596 17486113

[11] Chen X, Ba Y, Ma L, Cai X, Yin Y, Wang K, et al. Characterization of microRNAs in serum: a novel class of biomarkers for diagnosis of cancer and other diseases. Cell Res. 2008;18(10):997–1006. doi: 10.1038/cr.2008.282 18766170

[12] Tanaka T, Haneda S, Imakawa K, Sakai S, Nagaoka K. A microRNA, miR-101a, controls mammary gland development by regulating cyclooxygenase-2 expression. Differentiation. 2009;77(2):181–7. doi: 10.1016/j.diff.2008.10.001 19281778

[13] Kosaka N, Izumi H, Sekine K, Ochiya T. microRNA as a new immune-regulatory agent in breast milk. Silence. 2010;1(1):7. doi: 10.1186/1758-907X-1-7 20226005 PMC2847997

[14] Bozack AK, Colicino E, Rodosthenous RS, Bloomquist TR, Baccarelli AA, Wright RO, et al. Breast milk-derived extracellular vesicle miRNAs are associated with maternal asthma and atopy. Epigenomics. 2022;14(12):727–39. doi: 10.2217/epi-2022-0090 35638388 PMC9280402

[15] Kusuma RJ, Manca S, Friemel T, Sukreet S, Nguyen C, Zempleni J. Human vascular endothelial cells transport foreign exosomes from cow’s milk by endocytosis. Am J Physiol Cell Physiol. 2016;310(10):C800-7. doi: 10.1152/ajpcell.00169.2015 26984735 PMC4895447

[16] Cendron F, Rosani U, Franzoi M, Boselli C, Maggi F, De Marchi M, et al. Analysis of miRNAs in milk of four livestock species. BMC Genomics. 2024;25(1):859. doi: 10.1186/s12864-024-10783-4 39277740 PMC11401297

[17] Genty G, Sandoval-Castillo J, Beheregaray LB, Möller LM. Into the Blue: exploring genetic mechanisms behind the evolution of baleen whales. Gene. 2024;929:148822. doi: 10.1016/j.gene.2024.148822 39103058

[18] Reidenberg JS, Hanke FD. Marine mammal sensory systems: special issue overview. Anat Rec (Hoboken). 2022;305(3):509–13. doi: 10.1002/ar.24865 35077022

[19] Oftedal OT. Lactation in whales and dolphins: evidence of divergence between baleen- and toothed-species. J Mammary Gland Biol Neoplasia. 1997;2(3):205–30. doi: 10.1023/a:1026328203526 10882306

[20] La Manna G, Ronchetti F, Sarà G, Ruiu A, Ceccherelli G. Common bottlenose dolphin protection and sustainable boating: species distribution modeling for effective coastal planning. Front Mar Sci. 2020;7. doi: 10.3389/fmars.2020.542648

[21] Segawa T, Kobayashi Y, Inamoto S, Suzuki M, Endoh T, Itou T. Identification and expression profiles of microRNA in dolphin. Zoolog Sci. 2016;33:92–7. doi: 10.2108/zs150090 26853874

[22] Clarke AW, Høye E, Hembrom AA, Paynter VM, Vinther J, Wyrożemski Ł, et al. MirGeneDB 3.0: improved taxonomic sampling, uniform nomenclature of novel conserved microRNA families and updated covariance models. Nucleic Acids Res. 2025;53(D1):D116–28. doi: 10.1093/nar/gkae1094 39673268 PMC11701709

[23] Jang H, Park J, Kim VN. ERH promotes primary microRNA processing beyond cluster assistance. Nat Commun. 2025;16(1):7913. doi: 10.1038/s41467-025-63015-y 40854975 PMC12379101

[24] Han J, Pedersen JS, Kwon SC, Belair CD, Kim Y-K, Yeom K-H, et al. Posttranscriptional crossregulation between Drosha and DGCR8. Cell. 2009;136(1):75–84. doi: 10.1016/j.cell.2008.10.053 19135890 PMC2680683

[25] Chang T, Hancks DC. A subclass of small RNAs is encoded by exons of protein-coding genes. BMC Genomics. 2025;26(1):827. doi: 10.1186/s12864-025-11982-3 41013174 PMC12465437

[26] Wahid F, Shehzad A, Khan T, Kim YY. MicroRNAs: synthesis, mechanism, function, and recent clinical trials. Biochim Biophys Acta BBA - Mol Cell Res. 2010;1803:1231–43. doi: 10.1016/j.bbamcr.2010.06.013 20619301

[27] Mainieri A, Haig D. Retrotransposon gag-like 1 (RTL1) and the molecular evolution of self-targeting imprinted microRNAs. Biol Direct. 2019;14(1):18. doi: 10.1186/s13062-019-0250-0 31640745 PMC6805670

[28] Iqbal A, Yu H, Jiang P, Zhao Z. Deciphering the key regulatory roles of KLF6 and Bta-miR-148a on milk fat metabolism in bovine mammary epithelial cells. Genes (Basel). 2022;13(10):1828. doi: 10.3390/genes13101828 36292712 PMC9602136

[29] Hicks SD, Chandran D, Confair A, Ward A, Kelleher SL. Human milk-derived levels of let-7g-5p may serve as a diagnostic and prognostic marker of low milk supply in breastfeeding women. Nutrients. 2023;15(3):567. doi: 10.3390/nu15030567 36771276 PMC9920885

[30] van Herwijnen MJC, Driedonks TAP, Snoek BL, Kroon AMT, Kleinjan M, Jorritsma R. Abundantly present miRNAs in milk-derived extracellular vesicles are conserved between mammals. Front Nutr. 2018;5:81. doi: 10.3389/fnut.2018.00081 30280098 PMC6153340

[31] Do DN, Li R, Dudemaine PL, Ibeagha-Awemu EM. MicroRNA roles in signalling during lactation: an insight from differential expression, time course and pathway analyses of deep sequence data. Sci Rep. 2017;7:44605. doi: 10.1038/srep44605 28317898 PMC5357959

[32] Wang H, Zhu J, He Q, Loor JJ, Luo J. Association between the expression of miR-26 and goat milk fatty acids. Reprod Domest Anim. 2018;53(6):1478–82. doi: 10.1111/rda.13291 30058225

[33] Dall’Olio E, De Rensis F, Martignani E, Miretti S, Ala U, Cavalli V, et al. Differential Expression of miR-223-3p and miR-26-5p According to Different Stages of Mastitis in Dairy Cows. Biomolecules. 2025;15: 235. doi: 10.3390/biom15020235 40001538 PMC11853211

[34] Bian Y, Lei Y, Wang C, Wang J, Wang L, Liu L, et al. Epigenetic regulation of miR-29s affects the lactation activity of dairy cow mammary epithelial cells. J Cell Physiol. 2015;230(9):2152–63. doi: 10.1002/jcp.24944 25656908

[35] Li Q, Yang C, Du J, Zhang B, He Y, Hu Q, et al. Characterization of miRNA profiles in the mammary tissue of dairy cattle in response to heat stress. BMC Genomics. 2018;19(1):975. doi: 10.1186/s12864-018-5298-1 30593264 PMC6309072

[36] Menjivar NG, Gebremedhn S, Tesfaye D. 108 Preferential loading of thermal stress-associated microRNAs into extracellular vesicles: attempt to mitigate effects of heat stress in bovine granulosa cells. Reprod Fertil Dev. 2021;34(2):291. doi: 10.1071/RDv34n2Ab108 35231244

[37] Wu J, He D, Yue B, Zhang C, Fang X, Chen H. miR-101-1 expression pattern in Qinchuan cattle and its role in the regulation of cell differentiation. Gene. 2017;636:64–9. doi: 10.1016/j.gene.2017.09.026 28919162

[38] Yi J, Huang W-Z, Wen Y-Q, Yi Y-C. Effect of miR-101 on proliferation and oxidative stress-induced apoptosis of breast cancer cells via Nrf2 signaling pathway. Eur Rev Med Pharmacol Sci. 2019;23(20):8931–9. doi: 10.26355/eurrev_201910_19291 31696480

[39] Guo Y, Wu X, Zhen H, Feng Y, Li M, Ren C, et al. MicroRNA-30a-3p influences milk fat metabolism by targeting PTEN in mammary epithelial cells of sheep. Animals (Basel). 2025;15(8):1180. doi: 10.3390/ani15081180 40282014 PMC12023941

[40] Yang J, Hu QC, Wang JP, Ren QQ, Wang XP, Luoreng ZM, et al. RNA-Seq reveals the role of miR-29c in regulating inflammation and oxidative stress of bovine mammary epithelial cells. Front Vet Sci. 2022;9:865415. doi: 10.3389/fvets.2022.865415 35433915 PMC9011060

[41] Schanzenbach CI, Kirchner B, Ulbrich SE, Pfaffl MW. Can milk cell or skim milk miRNAs be used as biomarkers for early pregnancy detection in cattle?. PLoS One. 2017;12:e0172220. doi: 10.1371/journal.pone.0172220 PMC5325256 28234939

[42] Chu M, Zhao Y, Yu S, Hao Y, Zhang P, Feng Y, et al. miR-15b negatively correlates with lipid metabolism in mammary epithelial cells. Am J Physiol Cell Physiol. 2018;314(1):C43–52. doi: 10.1152/ajpcell.00115.2017 28835435

[43] Umu SU, Paynter VM, Trondsen H, Buschmann T, Rounge TB, Peterson KJ, et al. Accurate microRNA annotation of animal genomes using trained covariance models of curated microRNA complements in MirMachine. Cell Genom. 2023;3(8):100348. doi: 10.1016/j.xgen.2023.100348 37601971 PMC10435380

[44] Fromm B. A renaissance of microRNAs as taxonomic and phylogenetic markers in animals. Zool Scr. 2024;53:754–62. doi: 10.1111/zsc.12684

[45] Horreo JL. New insights into the phylogenetic relationships among the oceanic dolphins (Cetacea: Delphinidae). J Zool Syst Evol Res. 2019;57:476–80. doi: 10.1111/jzs.12255

[46] Tarver JE, Taylor RS, Puttick MN, Lloyd GT, Pett W, Fromm B. Well-annotated microRNAomes do not evidence pervasive miRNA loss. Genome Biol Evol. 2018;10:1457–70. doi: 10.1093/gbe/evy096 29788279 PMC6007596

[47] Langschied F, Leisegang MS, Günther S, Hahner F, Brandes RP, Ebersberger I. Loss of multiple micro-RNAs uncovers multi-level restructuring of gene regulation in rodents. BMC Genomics. 2025;26(1):800. doi: 10.1186/s12864-025-11815-3 40898033 PMC12403560

[48] Chen X, Song QL, Ji R, Wang JY, Li ZH, Guo D, et al. MiR-187 regulates the proliferation, migration and invasion of human trophoblast cells by repressing BCL6-mediated activation of PI3K/AKT signaling. Placenta. 2022;118:20–31. doi: 10.1016/j.placenta.2022.01.001 35007926

[49] Wang Z-S, Zhong M, Bian Y-H, Mu Y-F, Qin S-L, Yu M-H, et al. MicroRNA-187 inhibits tumor growth and invasion by directly targeting CD276 in colorectal cancer. Oncotarget. 2016;7(28):44266–76. doi: 10.18632/oncotarget.10023 27329595 PMC5190094

[50] Xu W, Liu W, Anwaier A, Tian X, Su J, Shi G, et al. Deciphering the role of miR-187-3p/LRFN1 axis in modulating progression, aerobic glycolysis and immune microenvironment of clear cell renal cell carcinoma. Discov Oncol. 2022;13(1):59. doi: 10.1007/s12672-022-00523-z 35799072 PMC9263027

[51] Tejada-Martinez D, de Magalhães JP, Opazo JC. Positive selection and gene duplications in tumour suppressor genes reveal clues about how cetaceans resist cancer. Proc Biol Sci. 2021;288(1945):20202592. doi: 10.1098/rspb.2020.2592 33622125 PMC7935004

[52] França GS, Hinske LC, Galante PAF, Vibranovski MD. Unveiling the impact of the genomic architecture on the evolution of vertebrate microRNAs. Front Genet. 2017;8:34. doi: 10.3389/fgene.2017.00034 28377786 PMC5359303

[53] Alsaweed M, Lai CT, Hartmann PE, Geddes DT, Kakulas F. Human milk miRNAs primarily originate from the mammary gland resulting in unique miRNA profiles of fractionated milk. Sci Rep. 2016;6:20680. doi: 10.1038/srep20680 26854194 PMC4745068

[54] Cendron F, Franzoi M, De Marchi M, Rosani U, Penasa M. Characterization of microRNA in cow milk and colostrum. J Dairy Sci. 2025;108(3):2981–94. doi: 10.3168/jds.2024-25145 39647631

[55] Carrillo-Lozano E, Sebastián-Valles F, Knott-Torcal C. Circulating microRNAs in breast milk and their potential impact on the infant. Nutrients. 2020;12(10):3066. doi: 10.3390/nu12103066 33049923 PMC7601398

[56] Yi J, Huang W-Z, Wen Y-Q, Yi Y-C. Effect of miR-101 on proliferation and oxidative stress-induced apoptosis of breast cancer cells via Nrf2 signaling pathway. Eur Rev Med Pharmacol Sci. 2019;23(20):8931–9. doi: 10.26355/eurrev_201910_19291 31696480

[57] Hu Y, Deng J, Tian K, Yang W-R, Luo N-J, Lian Y, et al. MiR-8-3p regulates hyperthermia-induced lactate secretion by targeting PPP2R5B in boar Sertoli cells. Mol Reprod Dev. 2019;86(11):1720–30. doi: 10.1002/mrd.23265 31489750

[58] Challis R, Kumar S, Sotero-Caio C, Brown M, Blaxter M. Genomes on a Tree (GoaT): a versatile, scalable search engine for genomic and sequencing project metadata across the eukaryotic tree of life. Wellcome Open Res. 2023;8:24. doi: 10.12688/wellcomeopenres.18658.1 36864925 PMC9971660

[59] Martin M. Cutadapt removes adapter sequences from high-throughput sequencing reads. EMBnet.journal. 2011;17:10–2. doi: 10.14806/ej.17.1.200

